# Notes and News

**Published:** 1899-05-20

**Authors:** 


### Notes and News.

(Collected and Communicated.)

### HOSPITAL MEETINGS.

Hospital for Consumption, Brompton.—Annual Meeting at four p.m., on Thursday, May 25th.

The service dinner of the Indian Medical Service will take place at the Hotel Cecil on Thursday, June 8th, Surgeon-General Sir William Guyer Hunter in the chair.

It is stated that the Vienna Academy of Medicine have censured Dr. Schenk for having published his theory as to the production of sex in the lay instead of the medical press in the first instance.

According to the last report of the Director of the Assistance Publique of Paris that city is sadly deficient in hospital accommodation. He states that the population requiring hospital assistance has increased, whilst the number of beds available have remained stationary.

Dr. Alexander Crum Brown will deliver the Harveian Oration at the Harveian Festival which will be held on June 2nd in the hall of the Royal College of Physicians, Edinburgh. The subject of the oration will be Dr. John Mayow.

A new Dispensary Bill has been passed in New York. This provides, amongst other clauses, that all dispensaries shall be licensed by the State Board of Charities, which is required to draw up rules and regulations for the conduct of such institutions and for those who make use of them. Any infringement is punishable by a fine.

A dinner is to be given to Sir William MacCormac by the house surgeons of St. Thomas's Hospital who held office during the time he was actively connected with the hospital and medical school. The dinner, which is to be held at the Café Royal, on Wednesday, May 24th, is to celebrate the third year of the tenure of the office of President of the Royal College of Surgeons by Sir William MacCormac. The chair will be taken by Mr. G. C. Franklin, senior house surgeon on the list.

An Italian Congress of Hygiene will be held at Como this year. An exhibition of hygienic appliances will be held during the congress.

The date for the opening of the new building of the Royal London Ophthalmic Hospital, in the City Road, by the Duke and Duchess of York, is Monday, June 26th.

The Honourable Walter Rothschild, M.P., will preside at the annual festival dinner, in aid of the funds of the City of London Hospital for Diseases of the Chest, to be held at the Criterion, on Wednesday, June 7th.

A wealthy resident of the United States has stated his intention of showing his gratitude to the Johns Hopkins Hospital by bequeathing to it his body for the furtherance of medical knowledge.

The Royal Institute of Public Health will hold its annual dinner at the Hotel Cecil on Wednesday, June 7th, when the Harben Prize will be presented by Lord Lister.

The Brenbry Inebriate Reformatory is to be enlarged for the reception of a greater number of inmates. In the meanwhile a building at Horfield is to be used as a reception house for 25 females.

The "Basilisk" arrived at Plymouth on Sunday flying the yellow flag. The port sanitary authorities made an inquiry, and were satisfied that the infection had not spread from the one case of yellow fever which had occurred, and the officers and men were consequently allowed to land.

In answer to a question in the House of Commons by Mr. Ascroft, respecting the deaths from enteric fever of soldiers belonging to the Berkshire Regiment, Mr. Wyndham stated that in that portion of the battalion stationed at King William's Town there had been 18 cases but no deaths, while among those stationed at Grahamstown there had been seven cases and six deaths.

By her will Mrs. Sarah Beach, of 4, James' Street, Westbourne Terrace, has bequeathed £300 each to the British Home for Incurables, the British Lying-in Hospital, the Cancer Hospital (Fulham Road), the Charing Cross Hospital, the Chelsea Hospital for Women (Queen's Elm), the City of London Hospital for Diseases of the Chest (Victoria Park), the Friedenheim Home of Peace, the Hospital and Home for Incurable Children (Maida Vale), the Hospital for Consumption and Diseases of the Chest, the Hospital for Sick Children (Great Ormond Street), the Hospital for Women (Soho Square), King's College Hospital, the London Hospital, the London Temperance Hospital, the Middlesex Hospital, the North London Consumption Hospital, the Paddington Green Children's Hospital, the Royal Orthopædic Hospital (Oxford Street), the Royal London Ophthalmic Hospital (Moorfields), St. George's Hospital (Hyde Park), the Seamen's Hospital Society (Greenwich), the Surgical Aid Society, the University College Hospital, the Western Ophthalmic Hospital, and the Westminster Hospital.

